# National nutrition strategies that focus on maternal, infant, and young child nutrition in Southeast Asia do not consistently align with regional and international recommendations

**DOI:** 10.1111/mcn.12937

**Published:** 2020-06-30

**Authors:** Tuan T. Nguyen, Ashley Darnell, Amy Weissman, Jennifer Cashin, Mellissa Withers, Roger Mathisen, Karin Lapping, Timothy D. Mastro, Edward A. Frongillo

**Affiliations:** ^1^ Alive & Thrive Southeast Asia FHI 360 Hanoi Vietnam; ^2^ Asia Pacific Regional Office (APRO) FHI 360 Bangkok Thailand; ^3^ Alive & Thrive Southeast Asia FHI 360 Yangon Myanmar; ^4^ Keck School of Medicine University of Southern California Los Angeles California USA; ^5^ Alive & Thrive FHI 360 Washington District of Columbia USA; ^6^ Chief Science Office, FHI 360 Durham North Carolina USA; ^7^ Arnold School of Public Health University of South Carolina Columbia, South Carolina USA

**Keywords:** ASEAN, maternal, infant, and young child nutrition (MIYCN), national nutrition strategy, plan of action for nutrition, Southeast Asia

## Abstract

We examined the consistency of national nutrition strategies and action plans (NNS) focusing on maternal, infant, and young child nutrition in Southeast Asia with regional and international recommendations. Between July and December 2017, we identified and extracted information on context, objectives, interventions, indicators, strategies, and coordination mechanisms from the most recent NNS in nine Southeast Asian countries. All NNS described context, objectives, and the following interventions: antenatal care, micronutrient supplementation during pregnancy, breastfeeding promotion, improved complementary feeding, nutrition in emergencies, and food fortification or dietary diversity. Micronutrient supplementation for young children was included in eight NNS; breastfeeding promotion during pregnancy and support at birth in seven; and school feeding, deworming, and treatment of severe acute malnutrition in six. All NNS contained programme monitoring and evaluation plans with measurable indicators and targets. Not all NNS covered wasting, exclusive breastfeeding, low birthweight, and childhood overweight. Strategies for achieving NNS goals and objectives were health system strengthening (nine), social and behaviour change communication (nine), targeting vulnerable groups (eight), and social or community mobilization (four). All addressed involvement, roles and responsibilities, and collaboration mechanisms among sectors and stakeholders. There was a delay in releasing NNS in Indonesia, Myanmar, and the Philippines. In conclusion, although Southeast Asian NNS have similarities in structure and contents, some interventions and indicators vary by country and do not consistently align with regional and international recommendations. A database with regularly updated information on NNS components would facilitate cross‐checking completeness within a country, comparison across countries, and knowledge sharing and learning.

**Abbreviations**
ASEAN: Association of Southeast Asian Nations
ICN: International Conference on Nutrition
NNS: National Nutrition Strategy or Action Plan
WHO: World Health Organization

**Key messages**

- National nutrition strategies or action plans (NNS) were available in nine of the 11 countries in Southeast Asia.
- The structure and some contents of the NNS were typically aligned with the First International Conference on Nutrition in 1992.
- NNS interventions and indicators vary by country and do not consistently align with regional and international recommendations for all countries.
- Reviewed NNS were not flexible for changes that limited their adaptability to new recommendations and might cause delay of releasing updated NNS.
- Establishing a database of nutrition strategy components would help to facilitate cross‐checking completeness within a country, comparison across countries, and knowledge sharing and learning.

## 1 INTRODUCTION

Most countries in the world are facing multiple and overlapping burdens of malnutrition (Development Initiatives, 2018). More than 30% of children are stunted, wasted, or overweight, and 50% suffer from hidden hunger due to deficiencies in vitamins and other essential nutrients (UNICEF, 2019). The triple burden of malnutrition harms children, adolescents, and women in the short and long term as well as negatively affects a country's economic and social development (UNICEF, 2019). Progress towards the Global Nutrition Targets 2025 and Sustainable Development Goals by 2030 is slow, especially for nutrition‐related chronic diseases and maternal anaemia (Development Initiatives, 2018; UNICEF, 2019).

Countries in Southeast Asia have high burdens of malnutrition (Association of Southeast Asian Nations [ASEAN], European Union, UNICEF, & World Health Organization [WHO], 2016; UNICEF, 2019). For example, in the region's 11 countries, nine have a high or very high prevalence of stunting (≥20%), nine have medium, high, or very high prevalence of wasting (≥5%), and five countries have medium, high, or very high prevalence of overweight (≥5%) among children under 5 years of age (UNICEF, 2019). The prevalence of children under 5 suffering from micronutrient deficiencies in Southeast Asia is almost 50% (ASEAN et al., 2016; UNICEF, 2019). Rates of malnutrition among school‐aged children and women are also high in this region (UNICEF, 2019). Seven of the 11 Southeast Asian countries belong to the lowest 20th percentile of height for men and women among 129 countries (N. C. D. Risk Factor Collaboration, 2016). This study also showed that men and women in Southeast Asia experienced a low increment in height between 1896 and 1996 (N. C. D. Risk Factor Collaboration, 2016). In addition, countries in this region are facing emerging issues related to health disparity, poor water and sanitation, food insecurity, climate change, globalization and urbanization, and sustainable agriculture production (ASEAN et al., 2016; UNICEF, 2019).

Comprehensive policy is recognized as a critical component of a national response to the triple burden of malnutrition (Food and Agriculture Organization of the United Nations [FAO] & WHO, 1992; WHO, 2018a). To achieve the goal of ending all forms of malnutrition, since the 1990s starting with the First International Conference on Nutrition (ICN) in 1992, countries have adopted national nutrition strategies and action plans (NNS; FAO & WHO, 1992; WHO, 2018a). The NNS provide governments with a framework for improving nutrition and food security by identifying the goals, objectives, interventions, resources, and roles and responsibilities of different stakeholders. To further address specific needs, priorities, and resources in Southeast Asia, there were several regional frameworks produced, including the WHO Southeast Asia's Addressing Malnutrition and Micronutrient Deficiencies (WHO South‐East Asia, 2011) and UNICEF East Asia Pacific's Strategic Approach and Implementation Guidance (UNICEF, 2014). The ASEAN member states also agreed on a regional framework and strategic plan to *End all Forms of Malnutrition* (ASEAN, 2017).

To provide an overview of national nutrition policies, plans of action, and programmes, WHO conducted a *Global Nutrition Policy Reviews* 2009–2010 and 2016–2017 (WHO, 2013, 2018a). The reviews found that most countries reported having national nutrition policies and plans of action and programmes to address undernutrition, obesity, diet‐related chronic diseases, infant and young child feeding, and vitamin and mineral malnutrition, yet there were also identified gaps in their design, content, and implementation (WHO, 2013, 2018a). The findings, however, were aggregated by WHO region (Southeast Asian countries were grouped in the South‐East Asia Region and Western Pacific Region), resulting in the unavailability of detailed information by country (WHO, 2013, 2018a). The *Report on Nutrition Security in ASEAN* (ASEAN et al., 2016) presents nutrition‐related issues covered by national policies in each of its 10 member countries. Other aspects of the policies, however, such as coverage of implementation, governance and partners, resources and capacity, and monitoring and evaluation, were not reported by country (ASEAN et al., 2016). In addition, because the information was updated by member countries based on all national policies, we do not know how well the NNS covered the context, goals, targets, commitment, and monitoring and evaluation (ASEAN et al., 2016).

It is well established that if evidence‐based interventions for women and children and evidence‐based nutrition interventions for the whole population are implemented with supportive policies and legislation and functioning health, education, and social protection systems, we can improve nutrition and health status of women and children, which contributes to economic development and increased equity (Bhutta et al., 2008; Bhutta et al., 2013; Ruel, Alderman, Maternal, & Child Nutrition Study, 2013; UNICEF, 2019). An NNS can serve as a guide for such multisectoral actions and act as a reference point for nutrition actions across other policies in health and other sectors. Although it is expected that an effective NNS would be aligned with international recommendations, few studies have been conducted to evaluate this aspect of NNS in Southeast Asia. To address this gap, we examined the alignment of NNS focusing on maternal, infant, and young child nutrition in Southeast Asia with regional and international recommendations.

### 1.1 Subjects and methods

**Figure 1:**
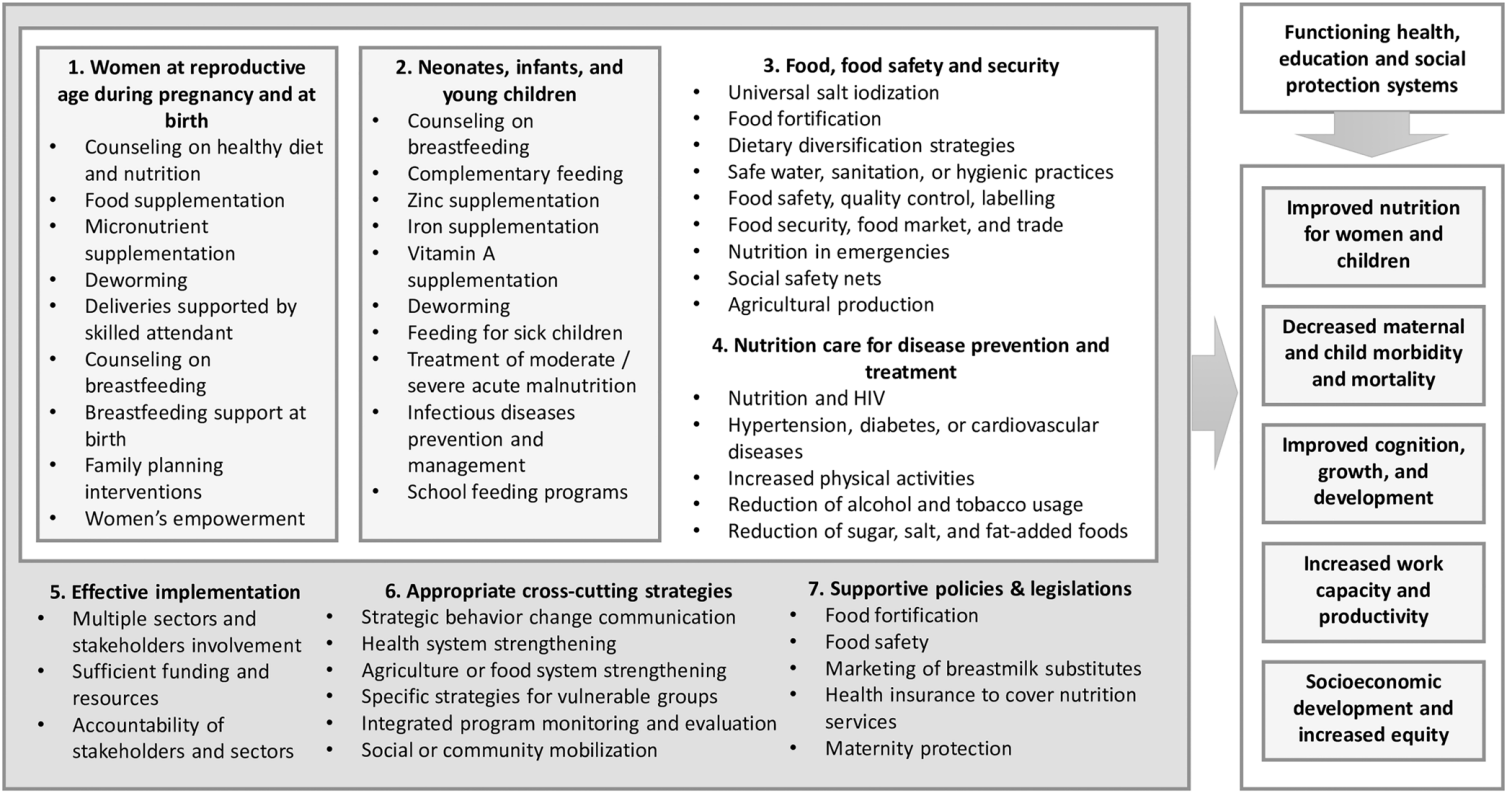
Conceptual framework

In a desk review of NNS focusing on MIYCN in Southeast Asian countries, we (a) identified NNS, (b) developed a quantitative data extraction form, (c) extracted information and managed the data, and (d) performed data analysis. Our study was based on a conceptual framework (Figure 1) that was developed on the basis of previous literature (Bhutta et al., 2008; FAO & WHO, 1992, 2014; Hausmann, Tyson, & Zahidi, 2006; Ruel et al., 2013; UNICEF, 2014, 2019; United Nations, 2017b).

#### 1.1.1 Policy identification

We looked for the most current NNS (as of December 31, 2017) from 11 Southeast Asian countries: Brunei, Cambodia, Indonesia, Laos, Malaysia, Myanmar, the Philippines, Singapore, Thailand, Timor‐Leste, and Vietnam. We obtained the full text of the NNS from the WHO Nutrition Information System (Nutrition Landscape Information System; WHO, 2018b), FAOLEX database (FAO, 2018), government websites, or personal contact with in‐country experts. We identified national nutrition policies, strategies, or plan of actions relating to MIYCN in nine countries (Government of Brunei, 2014; Government of Cambodia, 2014; Government of Indonesia, 2011; Government of Laos, 2015; Government of Malaysia, 2016; Government of Myanmar, 2013; Government of the Philippines, 2012; Government of Vietnam, 2012; Ministry of Health Timor‐Leste, 2014). As of December 31, 2017, a newer, full‐text policy for Indonesia, Myanmar, and the Philippines had not been released, so we used the older versions: *Indonesia National Food and Nutrition Action Plan 2011–2015* (Government of Indonesia, 2011), *Myanmar National Plan of Action for Food and Nutrition 2011–2015* (Government of Myanmar, 2013), and *Philippines Plan of Action for Nutrition 2011–2016* (Government of the Philippines, 2012). Singapore's *Healthy Living Master Plan* with vision to 2020 (Government of Singapore, 2014) and Thailand's *Healthy Lifestyle Strategic Plan* *2011–* *2020* and *National Health Development Plan* *2017–* *2021* (Government of Thailand, 2011) were not part of the analysis because they focused on overall health and lifestyle with limited information about MIYCN. In addition, their structure and contents were not comparable with the other nine countries' NNS that typically followed the conventional ICN guidelines (FAO & WHO, 1992).

#### 1.1.2 Data extraction form

We developed a form that covered general characteristics of NNS (policy name, year published, publisher, year of adoption, adoptee, and start and end year); categories: material (to result in actual changes) and symbolic (to articulate aspirations for social betterment); governing resources: information or knowledge (to educate or change behaviour of policy targets); authority (to regulate); treasury (to specify the availability and use of financial resources); and organization structure (to stipulate tasks to be done by relevant sectors or stakeholders; Shroff, Jones, Frongillo, & Howlett, 2012). The contents included policy context, goals, objectives, strategies, interventions, implementation, monitoring and evaluation, and sectors and stakeholders involved in the policies as well as their roles and collaboration mechanisms. The list of interventions in the extraction form was based on those in the ICN 1992 and 2014 (FAO & WHO, 1992, 2014), the Lancet Series on Maternal and Child Undernutrition (Bhutta et al., 2008), and the Lancet Series on Maternal and Child Nutrition (Bhutta et al., 2013; Ruel et al., 2013). The list of indicators was based on relevant indicators from Global Nutrition Targets 2025, Sustainable Development Goals, Millennium Development Goals, ICN 1992, and ICN 2014 (FAO & WHO, 1992, 2014;United Nations, 2008, 2017a ; WHO, 2017).

#### 1.1.3 Data extraction and management

We extracted NNS data using the hardcopy data extraction form. Number‐coded information was then inputted into a Microsoft Excel data entry form. The NNS were reviewed by one researcher and cross‐checked by another. We used PowerQuery in Microsoft Excel for data management and PowerPivot for data analysis and presentation.

#### 1.1.4 Data analysis

We provided descriptive findings of each NNS and compared its structure, interventions, and indicators with those from other NNS and previous studies or guidelines (Bhutta et al., 2008; Bhutta et al., 2013; FAO & WHO, 1992, 2014; Ruel et al., 2013). We applied the effectiveness ranking for select interventions (e.g., strong evidence, mixed evidence, and weak evidence) produced by previous studies (Bhutta et al., 2008; Bhutta et al., 2013; Ruel et al., 2013; Webb & Kennedy, 2014). Indicators were cross‐referenced with the Global Nutrition Targets, the Millennium Development Goals, and the Sustainable Development Goals (United Nations, 2008, 2017a; WHO, 2017).

## 2 RESULTS

Of the 11 Southeast Asian countries, nine had approved NNS as a national guiding document to provide information knowledge, organization structure, and type of instrument (Table 1). Five NNS have a treasury governing resource cited, seven have mixed, and two have voluntary legal binding (Table 1). Although the nine Southeast Asian NNS were aligned with the structure proposed by the ICN in 1992, there was considerable variation in the level of detail included. Each NNS clearly outlined the country context in which the policy was developed (Table S1).

**Table 1:** Characteristics of national nutrition strategies reviewed, by country

Nutrition strategies	Approved by government	Material policy instrument	Governing resources				No. of pages
			Information, knowledge	Authority	Treasury	Organization structure	
Brunei National Strategy for Maternal, Infant, and Young Child Nutrition (2014–2018)	√	√	√	√		√	78
Cambodia National Strategy for Food Security and Nutrition (2014–2018)	√	√	√	√	√	√	94
Indonesia National Food and Nutrition Action Plan (2011–2015)	√	√	√	√	√	√	83
Laos National Nutrition Strategy to 2025 and Plan of Action (2016–2020)	√	√	√	√	√	√	73
Malaysia National Plan of Action for Nutrition (2016–2025)	√	√	√	√	√	√	176
Myanmar National Plan of Action for Food and Nutrition (2011–2015)	√	√	√	√		√	43
Philippines Plan of Action for Nutrition (2012–2015)	√	√	√	√		√	23
Timor‐Leste National Nutrition Strategy (2014–2019)	√	√	√	√		√	77
Vietnam National Nutrition Strategy (2011–2020)	√	√	√	√	√	√	48

*Note.* A newer policy for Indonesia, Myanmar, and the Philippines has not been released as of December 31, 2017. Categories of policy instrument: material (to result in changes in actual) and symbolic (to articulate aspirations for social betterment); governing resources: information or knowledge (to educate or change behaviour of policy targets); authority (to regulate); treasury (to specify the availability and its use of financial resources); and organization structure (to stipulate tasks to be done by relevant sectors or stakeholders).

NNS outlined interventions for women at reproductive age, including teenagers, during pregnancy such as dietary counselling (seven), deworming (five), and protein and energy and micronutrient supplementation (nine; Table 2). Breastfeeding promotion during pregnancy (using individual or group counselling) was not included in the NNS of Cambodia or Laos, and breastfeeding support at birth was not included in that of the Philippines or Vietnam (Table 2). Nutrition during the preconception period and for teenagers (other than during pregnancy) was not included in the majority of NNS.

**Table 2:** Interventions included in national nutrition strategies, by country

Interventions	Intervention effectiveness^a^	Brunei	Cambodia	Indonesia	Laos	Malaysia	Myanmar	Philippines	Timor‐Leste	Vietnam
Women at reproductive age during pregnancy and at birth										
Dietary counselling, keeping physically active, or tracking weight gain during pregnancy	3	√	√	√		√		√	√	√
Maternal supplementation of balanced energy and protein diets	1^b^	√	√	√	√	√	√	√	√	√
Maternal micronutrient supplementation during pregnancy (including iron folate, calcium, multiple micronutrients, or iodine)	1/1^b^	√	√	√	√	√	√	√	√	√
Deworming in pregnancy	1^b^				√		√	√	√	√
Deliveries supported by skilled attendant	NR						√		√	
Breastfeeding promotion during pregnancy (including individual and group counselling)	1	√		√		√	√	√	√	√
Breastfeeding support at birth (including essential newborn care and the Baby‐Friendly Hospital Initiative)	1^b^/2	√	√	√	√	√	√		√	
Women's empowerment and the prevention of domestic violence or gender‐based violence	1^b^/NR		√		√		√		√	
Family planning interventions to promote birth spacing	2			√	√		√	√	√	
Neonates, infants, and young children										
Breastfeeding promotion (individual and group counselling, including exclusive breastfeeding under 6 months and prolonged breastfeeding at 1 and 2 years)	1	√	√	√	√	√	√	√	√	√
Improved complementary feeding (including timely introduction of complementary foods, dietary diversity, and meal frequency)	1	√	√	√	√	√	√	√	√	√
Zinc supplementation (including those for diarrhoeal children)	1		√		√		√	√	√	
Iron supplementation	1^b^		√	√	√	√	√	√	√	√
Vitamin A supplementation	1		√	√	√	√	√	√	√	√
Deworming	1^b^		√		√		√	√	√	√
Feeding for sick children (including diarrhoea and respiratory infection)	NR	√			√			√	√	√
Treatment of moderate/severe acute malnutrition	1		√		√		√	√	√	√
Infectious diseases prevention and management (e.g., diarrhoea, acute respiratory infection, and malaria)	1/1^b^				√		√	√	√	√
School feeding programmes	3		√	√	√	√	√			√
Food, food safety, and food security										
Universal salt iodization	1		√	√	√	√	√	√	√	√
Food fortification (including vitamin A and iron)	1^b^		√	√	√	√	√	√	√	√
Dietary diversification strategies, small animal husbandry, or home gardening	2	√	√	√	√	√	√	√	√	√
Safe water, sanitation, or hygienic practices	1		√	√	√		√	√	√	√
Food safety, quality control, the prevention of food‐borne diseases, or food labelling	NR	√	√	√	√	√	√	√	√	√
Food security, food market, and trade	1^b^/NR		√	√	√	√	√	√	√	√
Nutrition in emergencies	1^b^	√	√	√	√	√	√	√	√	√
Social safety nets, cash transfers, microcredit programmes, food‐for‐work programmes, or generalized food subsidies	1^b^/NR		√	√		√	√	√	√	
Agricultural production subsidies, land use, or reform	1^b^/NR		√	√	√	√	√	√	√	√
Nutrition care for disease prevention and treatment										
Nutrition and HIV	1/1^b^	√	√		√		√		√	√
Hypertension, diabetes, or cardiovascular diseases	NR	√	√		√	√		√	√	√
Increased physical activities	NR			√		√		√		√
Reduction of alcohol consumption or tobacco usage	1			√		√	√	√		
Reduction of sugar and fat‐added foods, sweetened beverages, or salt consumption	NR					√	√	√		
Cross‐cutting strategies										
Mass communication	3		√			√	√	√	√	√
Interpersonal communication	1	√	√	√	√	√	√	√	√	√
Nutritional or health campaigns	3		√	√	√	√	√	√	√	√
Health system strengthening	NR	√	√	√	√	√	√	√	√	√
Agriculture or food system strengthening	1		√	√	√	√	√	√	√	√
Specific strategies for vulnerable groups	1/1^b^		√	√	√	√	√	√	√	√
Integrated programme monitoring and evaluation	1^b^	√	√	√	√	√	√	√	√	√
Social or community mobilization	NR				√		√		√	√
Supportive policies and legislations										
Strengthen legislations on food fortification	NR		c	c	c	c	c	c	c	c
Strengthen legislations on food safety	NR	√	√	√	c	√	√	√	c	√
Strengthen policies and commitments relating to food and nutrition or incorporating nutrition goals into relevant laws, regulations, policies, and plans	NR	√	√	√	√	√	√	√	√	√
Strengthen legislations on marketing of breastmilk substitutes	NR	√	√	√	√	√	√	√	√	
Health insurance to cover nutrition, curative care for young children, or nutrition preventative care	NR						√	√	√	
Strengthen legislations on maternity leave or workplace lactation support	NR	√	√			√		√		√

a Intervention effectiveness on maternal and child nutrition: (a) sufficient evidence, (b) insufficient or variable evidence, and (c) little or no evidence; NR, not reviewed (Bhutta et al., 2008; Ruel et al., 2013; Webb & Kennedy, 2014).

b Interventions effective in specific context.

c Under development.

Promotion of optimal breastfeeding and complementary feeding after the post‐natal period was included in nine NNS. Regarding nutrition interventions for children and the general population, micronutrient supplementation (e.g., zinc, iron, and vitamin A) was included in all but the NNS of Brunei. NNS from Brunei, Malaysia, and Indonesia did not include child deworming and treatment of severe acute malnutrition interventions among children. NNS from Brunei, the Philippines, and Timor‐Leste did not mention school feeding programmes (Table 2).

Interventions relating to food, food safety, and food security were commonly listed in the NNS: food diversity strategies (nine), food fortification interventions (eight), nutrition in emergencies (nine), food safety (nine), and provision of social safety nets (six; Table 2). Some NNS indicated relevant policies such as food fortification regulations (eight fortification policies under development), food safety (seven, and two under development), regulations on the marketing of breastmilk substitutes (eight), and maternity protection, including maternity leave and workplace lactation support (five; Table 2). Only a subset of NNS included nutrition and HIV (six), chronic diseases (seven), and healthy lifestyle (five).

Cross‐cutting strategies for achieving the goals and objectives of NNS were interpersonal communication (nine NNS), national health campaigns (eight), mass communication (six), health system strengthening (nine), agriculture or food system strengthening (eight), and interventions for vulnerable groups (eight; Table 2). NNS from Laos, Myanmar, Timor‐Leste, and Vietnam had social or community mobilization strategies. All NNS contained strategies for monitoring, evaluation, surveillance, and implementation of surveys to measure progress and impact (Table 2).

All nine NNS had indicators and targets relating to MIYCN. Indicators of infant and young child nutrition status included low birthweight (seven NNS), stunting (nine), wasting (eight), underweight (seven), overweight (six), iron deficiency anaemia (six), vitamin A deficiency (six), and iodine deficiency disorders (six). Indicators for breastfeeding practices included early initiation of breastfeeding (four), exclusive breastfeeding (eight), and continued breastfeeding at 1 or 2 years (four; Table 3). The NNS in Laos, Malaysia, Myanmar, and Timor‐Leste had at least three out of four recommended indicators relating to complementary feeding or dietary quality. Those from Cambodia and Vietnam had one indicator, whereas the remaining three countries had none. Indicators for nutrition status of women at reproductive age included iron deficiency anaemia (nine NNS), underweight (a body mass index <18.5 kg m^−2^; six), and overweight or obesity (five; Table 3).

**Table 3:** Nutrition indicators included in national nutrition strategies, by country

Indicators	GNTs 2025	MDGs 2015	SDGs 2030	Brunei	Cambodia	Indonesia	Laos	Malaysia	Myanmar	Philippines	Timor‐Leste	Vietnam
Infant and young child nutrition												
Low birthweight	√			√			√	√	√	√	√	√
Stunting	√		√	√	√	√	√	√	√	√	√	√
Wasting	√		√	√	√	√	√	√	√	√	√	
Underweight		√			√		√	√	√	√	√	√
Childhood overweight and obesity	√		√	√			√	√	√	√		√
Iron deficiency anaemia					√		√		√	√	√	√
Vitamin A deficiency					√	√		√		√	√	√
Iodine deficiency disorders							√	√	√	√	√	√
Early initiation of breastfeeding					√		√	√			√	
Exclusive breastfeeding	√			√	√	√	√	√	√		√	√
Continued breastfeeding at 1 and 2 years					√	√			√		√	
Timely introduction of complementary foods							√	√	√		√	
Minimum meal frequency							√	√				
Minimum dietary diversity							√	√	√		√	√
Minimum acceptable diet		√			√		√	√	√		√	
Nutrition status of women at reproductive age												
Iron deficiency anaemia	√			√	√	√	√	√	√	√	√	√
Chronic energy deficiency (BMI < 18.5 kg m^−2^)					√	√		√		√	√	√
Overweight and obesity					√			√	√	√		√

Abbreviations: BMI, body mass index; GNTs, World Health Assembly's Global Nutrition Targets; MDGs, United Nations' Millennium Development Goals; SDGs, United Nations' Sustainable Development Goals.

Each NNS outlined sector and stakeholder roles, responsibilities, collaborative mechanisms, and whether the strategy is executed through a focal sector, among sectors, or among stakeholders (Table 4). The roles of governmental stakeholders (at national and subnational levels) were measured by the contribution of financial resources, provision of technical support, and implementation (Table S2). Technical support was typically the role of international organizations, donors, the private sector, and academic or research institutions (Table S2). Each NNS presented this information differently and with varying detail. Brunei, Cambodia, Laos, Myanmar, Timor‐Leste, and Vietnam all had specific sections outlining the sectors or stakeholders. The Indonesia NNS named the sectors in the text as well as in a table but was not specific about roles. The Malaysia NNS had a section dedicated to those involved in implementation but did not specify to what extent. The Philippines NNS mentioned sectors and stakeholders throughout the text, but there was no specific section or table referencing roles.

**Table 4:** Sectors and stakeholders involved in national nutrition strategies, by country

Sectors and stakeholders	Brunei	Cambodia	Indonesia	Laos	Malaysia	Myanmar	Philippines	Timor‐Leste	Vietnam
Sectors involved									
Health and nutrition	√	√	√	√	√	√	√	√	√
Agriculture		√	√	√	√	√	√	√	√
Food industry		√	√	√	√	√	√	√	
Education	√		√	√	√	√	√	√	√
Culture, information, and communication	√			√	√				√
Science, technology, and environment				√	√	√		√	
Labour and social affairs	√	√	√	√			√	√	√
Finance				√		√	√	√	
Internal and external trade				√	√				
Planning and investment	√		√	√	√	√	√		√
Stakeholders involved^a^									
National level (including government, parliament, and ministries)	√	√	√	√	√	√	√	√	√
Subnational levels (including provincial, city, district, and subdistrict local authorities in various sectors such as civil, health, nutrition, education, and agriculture)		√	√	√	√	√	√	√	√
Civil society organizations or unions^b^	√	√	√	√				√	√
International organizations or donors^c^	√	√	√	√	√	√		√	√
Private sector	√	√	√		√		√	√	
Academic or research institutions	√	√	√		√	√		√	√
Contains section dedicated to sector and stakeholder involvement	√	√		√		√		√	√
Clearly describes the roles and responsibilities of sectors and stakeholders	√	√		√		√		√	√
Collaboration mechanism indicated	√	√	√	√	√	√	√	√	√

a Data that allow for specific contribution such as technical support, financial support, or implementation are included in Table S2.

Civil society organizations and unions include unions (trade, women, farmers, and youth), societies (veterans, teachers, and elderly), and religious, village, and tribe leaders.

International Organizations, donors include UNICEF, World Health Organization, Food and Agriculture Organization of the United Nations, World Bank, other development bank (e.g., Asian Development Bank), governments of other countries (e.g., United States Agency for International Development, Australian Aid, and UK Aid), foundations (e.g., Bill & Melinda Gates Foundation), research foundation, international non‐governmental organizations, and in‐country donors.

## 3 DISCUSSION

Our analysis found that although Southeast Asian NNS have strengths, limitations remain, which may hinder countries' ability to combat the triple burden of malnutrition and to improve health and well‐being of their populations. A key strength of NNS in this region is that their structure and contents were typically aligned with the ICN in 1992 (FAO & WHO, 1992), which allow users and readers to capture information and apply it. For more than 25 years, the logical framework of NNS has facilitated nutrition planning, implementation, monitoring, and evaluation, which help to improve nutrition and health status in the world (FAO & WHO, 1992, 2014).

Limitations include the long process of developing and obtaining high‐level government approval of an NNS, which may have delayed release in Indonesia, Myanmar, and the Philippines. Similarly, developing or revising an NNS requires progress and impact data from the previous 5–10 years, and such evidence requires a robust, streamlined, reliable electronic monitoring system for inputs, outputs, outcomes, and impacts, which may be lacking in some countries. Due to these factors, modifying approved NNS to adopt new interventions or indicators during implementation is typically not feasible. Although determining reasons for these delays was beyond the scope of this study, often policy development is affected by a lack of evidence, consensus, or champion (Baker et al., 2018; Pelletier et al., 2013).

Successful implementation of NNS required involvement of different stakeholders and sectors (FAO & WHO, 1992, 2014). Yet, similar to findings from the Global Nutrition Policy Report (WHO, 2018a), we found that not all NNS specified sector and stakeholder engagement or financial commitment. This gap would hinder governments' ability to hold stakeholders accountable for their role in achieving nutrition targets. Thus, nutrition and health sectors become the main players in nutrition programmes (WHO, 2018a), which would prevent the country or region from applying a comprehensive systems approach to effectively, efficiently, and sustainably promote nutrition and health status (UNICEF, 2019; WHO, 2018a).

Like findings from the Global Nutrition Policy Reviews (WHO, 2013, 2018a) and the *Report on Nutrition Security in ASEAN* (ASEAN et al., 2016), we found that key interventions and targets or indicators relating to MIYCN were included in the reviewed NNS. Evidence‐based, effective interventions included in all NNS were improved diet and micronutrient supplementation for pregnant women, breastfeeding promotion, improved complementary feeding and delivery of vitamin A, and iron supplementation (Bhutta et al., 2008). Not all NNS included effective interventions that have potential impact in Southeast Asian countries such as provision of deworming for pregnant women and children, infectious diseases prevention and treatment, feeding for sick children, responsive feeding, and treatment of moderate or severe acute malnutrition (ASEAN et al., 2016; UNICEF, 2019). Another limitation of the NNS was that most of them did not have interventions during the preconception period and for teenagers (other than during pregnancy). This limitation was reported earlier in this region and globally (ASEAN et al., 2016; Bhutta et al., 2013; WHO, 2018a). These populations should be a focus on both nutrition and nonnutrition interventions to ensure that women enter pregnancy when they are physically and psychologically ready and thus improve quality of pregnancy, reduction of complications, and improved birth outcomes (UNICEF, 2019).

Interventions to prevent micronutrient deficiencies for a country's population such as universal salt iodization, food fortification, and dietary diversification strategies, small animal husbandry, or home gardening were included in almost all NNS, but their implementation is uncertain. For example, food fortification legislation was only under development in the nine countries; legislation making universal salt iodization mandatory was not enacted in Brunei, Singapore, and Vietnam (ASEAN et al., 2016). In Vietnam, marked decreases in iodized salt consumptions and increases in iodized deficiency were observed after the revocation of national legislation for mandatory salt iodization in 2005 (Codling et al., 2015). Home gardening, animal husbandry, cash transfers, provision of credit, and land distribution were included in some NNS however have showed mixed results on improving nutrition (Webb & Kennedy, 2014). Despite the emergence and burden of obesity and chronic diseases in Southeast Asia (ASEAN et al., 2016; UNICEF, 2019), these topics were not a priority focus in NNS in Southeast Asia. This may be due to the historical focus on undernutrition (FAO & WHO, 1992; UNICEF, 2019). These findings highlight the need to support governments to redirect policy and resources towards evidence‐based interventions and integrate multisectoral interventions to achieve greater effect in the context of a triple burden of malnutrition (ASEAN et al., 2016; UNICEF, 2019).

Indicators and targets listed in NNS were mostly nutrition indicators. Compared with the list of six indicators of the Global Nutrition Targets (WHO, 2017), all countries tracked prevalence of stunting (under 5 years old), but other six other recommended indicators. This gap limits the utility of the NNS in tracking the region's contribution to progress towards the Global Nutrition Targets 2025. By contrast, the Global Nutrition Targets does not include key infant and young child feeding indicators (aside from exclusive breastfeeding under 6 months). This finding suggests the need to promote the use of an extended list of nutrition indicators including those for key infant and young child feeding as well as the more broad Sustainable Development Goal indicators (United Nations, 2017a) across sectors in countries and regions for the purpose of planning, monitoring, and evaluation.

Our study has implications for Southeast Asia and beyond. This study reviewed NNS in Southeast Asia, a region with a total land area of ~4.5 million km^2^, a population of ~650 million, an annual gross domestic product of nearly US$ 3 trillion, and close financial connections with other economies (World Bank, 2018). Southeast Asia also comprises countries with diverse socio‐economic development ranging from high income (Singapore), higher middle‐income (Brunei, Malaysia, and Thailand), to lower middle income (remaining six countries; World Bank, 2018). Within these categories of socio‐economic development, there are complex health, nutrition, and diseases patterns that influence the triple burden of malnutrition (Development Initiatives, 2018; Popkin, 2002; UNICEF, 2019). This review of NNS across Southeast Asian countries could help countries in this region ensure cohesiveness and comparability among themselves for their overall framework to address the triple burden in the region (ASEAN et al., 2016; UNICEF, 2019). Because globalization, regional labour force agreements, and migration within Southeast Asia and beyond are increasing (ASEAN, 2018), understanding the nutrition and health status, priorities, and policies of one country is important for health planning or adaptation in other countries within and between regions. Findings of this study could also help to facilitate ongoing efforts by ASEAN to develop a regional surveillance system for nutrition indicators among member states. The data extraction form, data entry form, data management and analysis tools, and data are publicly available to facilitate study replication and used by researchers who wish to examine existing nutrition policies in other regions or the longitudinal policy progress in a given country.

Our study has several limitations. First, we did not examine how the NNS were implemented and interacted with other policies, the costs and benefits of the interventions within, or stakeholders' perception of the policies. These topics were beyond the scope of this study. Evidence from South and Southeast Asian countries show that stakeholders' engagement in infant and young child feeding policy may vary substantially by country (Michaud‐Letourneau, Gayard, & Pelletier, 2019; Uddin et al., 2017). Second, although various platforms have been used for regional and global coordination for improving MIYCN such as the SUN movement, Zero Hunger, Nutrition for Growth, REACH, United Nations Global Nutrition Agenda, and the regional frameworks of UNICEF and WHO, we did not consider the contribution of these platforms. These, however, have been discussed previously (ASEAN et al., 2016; Nutrition for Growth, 2018; REACH, 2018; Scaling Up Nutrition, 2018; UNICEF, 2014; United Nations, 2015; WHO South‐East Asia, 2011; Zero Hunger, 2018). Third, there were limitations of analysing only a single NNS policy for each country, including the lack of NNS in two key ASEAN countries, Singapore and Thailand, creating a missed opportunity for a full regional comparative analysis. Additionally, the NNS is specific to MIYCN and therefore may not fully cover other aspects of nutrition and health. Nonetheless, the list of interventions from all relevant policies could be found in other policy reviews (ASEAN et al., 2016). Fourth, although all NNS were officially translated into English and released by the countries, there was a potential for information bias if there were any discrepancy between the English and the local language versions. To minimize this risk, NNS were reviewed and verified by at least two researchers or experts, and the findings were verified by our multidisciplinary team and in‐country experts.

In conclusion, although Southeast Asian NNS have similarities in structure and some contents, some interventions and indicators vary by country and do not consistently align with regional and international recommendations. Updating information on nutrition strategy components in a new or existing database would help to facilitate cross‐checking completeness within a country, comparison across countries, and knowledge sharing and learning. This effort would facilitate coordination of an evidence‐based, well‐measured policy framework with clearly defined roles and responsibilities for ending all forms of malnutrition in the region in the world.

## CONFLICTS OF INTEREST

The authors declare that they have no conflicts of interest.

## CONTRIBUTIONS

TTN, AD, MW, RM, KL, and EAF designed the research; TTN and AD acquired the data, reviewed the policies, and analysed the data; TTN, AD, and AW drafted and revised the manuscript; AW, JC, MW, RM, KL, TDM, and EAF reviewed and interpreted the findings and provided substantial feedback and revisions on the manuscript. All authors have read and approved the final manuscript and had responsibility for the final content.

## Supporting information


**Table S1.** The context and objectives indicated in the policies
**Table S2.** Specific roles of stakeholders involved in national nutrition strategies, by country
