# Supplementary material for: National nutrition strategies that focus on maternal, infant, and young child nutrition in Southeast Asia do not consistently align with regional and international recommendations
Source: Matern Child Nutr. 2020 Jun 30;16(Suppl 2):e12937. doi: 10.1111/mcn.12937 (PMC7591308; doi:10.1111/mcn.12937)
Supplement: Supplementary file 1 — Table S1. The context and objectives indicated in the policies Table S2. Specific roles of stakeholders involved in national nutrition strategies, by country [file MCN-16-e12937-s001.docx]

**Supplemental Table 1.** The context and objectives indicated in the policies

|  | Brunei | Cambodia | Indonesia | Laos | Malaysia | Myanmar | Philippines | Timor-Leste | Vietnam |
| --- | --- | --- | --- | --- | --- | --- | --- | --- | --- |
| Policy context |  |  |  |  |  |  |  |  |  |
| Poverty or economic problem |  | √ | √ | √ |  | √ | √ | √ | √ |
| Issues relating to agriculture, food production, food distribution, food insecurity, or hunger |  | √ | √ | √ | √ | √ | √ | √ | √ |
| Population related factors such as change or an aging population |  |  |  |  | √ | √ |  |  | √ |
| Health problem of select groups (including mortality or morbidity, low life-expectancy at birth, and HIV) |  |  | √ |  |  | √ | √ |  |  |
| Poor nutrition status of infants and young children | √ | √ | √ | √ | √ | √ | √ | √ |  |
| Poor nutrition status of adolescents, women, and mothers (including chronic energy malnutrition and short stature (height < 1.45 m)) | √ | √ | √ | √ | √ | √ | √ | √ | √ |
| Micronutrient deficiencies (e.g., Vitamin A, Vitamin D, zinc, iron, iodine) | √ | √ | √ | √ | √ | √ | √ | √ | √ |
| Nutrition-related chronic diseases (e.g., cardiovascular diseases and risks, including overweight, obesity, hypertension, and diabetes) | √ |  | √ | √ | √ | √ | √ |  | √ |
| Issues relating to education, gender, women’s rights, early marriage, or inequalities |  |  | √ |  |  | √ |  | √ | √ |
| Unsafe water or sanitation |  | √ | √ | √ |  | √ |  | √ |  |
| Environment, climate change, or natural disasters |  | √ |  |  |  |  | √ |  | √ |
| Factors relating to resources (including human resource, funding, and materials) |  |  |  | √ |  | √ | √ | √ | √ |
| Data were used to construct the studied strategies | √ | √ | √ | √ | √ | √ | √ | √ | √ |
| Policy objectives |  |  |  |  |  |  |  |  |  |
| To improve diet (e.g., quantity and quality) |  | √ | √ |  | √ | √ | √ | √ | √ |
| To improve the nutrition status of mothers and children (including focus on protein energy malnutrition) | √ | √ | √ | √ | √ | √ | √ | √ | √ |
| To improve micro-nutrient status (e.g., Iron, Zinc, Vitamin A, Vitamin D) | √ |  |  |  |  |  |  | √ | √ |
| To prevent and control overweight, obesity, or other chronic diseases | √ |  |  |  | √ |  |  |  | √ |
| To prevent and control of infectious disease (including foodborne and waterborne diseases) |  |  |  |  |  | √ |  | √ |  |
| To improve knowledge and practices regarding nutrition in the general population |  | √ |  |  |  | √ | √ | √ | √ |
| To strengthen the national or local health system, or to reinforce the capacity and effectiveness of the nutrition services network in both community and health care facilities |  |  | √ | √ |  | √ | √ | √ | √ |
| To reduce inequities or barriers in access to care or to reduce nutrition and health disparities |  | √ | √ | √ |  |  | √ |  |  |

**Supplemental Table 2.** Specific roles of stakeholders involved in national nutrition strategies, by country

|  | Brunei | Cambodia | Indonesia | Laos | Malaysia | Myanmar | Philippines | Timor-Leste | Vietnam |
| --- | --- | --- | --- | --- | --- | --- | --- | --- | --- |
| Financial resources |  |  |  |  |  |  |  |  |  |
| National level | √ | √ | √ | √ | √ | √ | √ | √ | √ |
| Sub-national levels |  |  |  |  |  |  |  |  | √ |
| Civil society organizations, or unions† |  |  |  |  |  |  |  |  |  |
| International organizations, or donors‡ |  |  |  |  | √ |  |  | √ | √ |
| Private sector |  |  | √ |  |  |  |  |  |  |
| Technical support |  |  |  |  |  |  |  |  |  |
| National level | √ | √ | √ | √ | √ | √ | √ | √ |  |
| Sub-national levels |  | √ |  | √ | √ | √ | √ | √ |  |
| Civil society organizations, or unions | √ |  | √ | √ |  |  |  | √ | √ |
| International organizations, or donors | √ | √ |  | √ | √ | √ |  | √ |  |
| Private sector | √ | √ |  |  | √ |  | √ | √ |  |
| Academic or research institutions | √ | √ | √ |  | √ | √ |  | √ | √ |
| Implementation |  |  |  |  |  |  |  |  |  |
| National level | √ | √ | √ | √ | √ | √ | √ |  | √ |
| Sub-national levels |  | √ | √ | √ | √ | √ | √ | √ | √ |
| Civil society organizations, or unions |  | √ | √ | √ |  |  |  | √ | √ |
| International organizations, or donors | √ |  |  |  | √ | √ |  |  |  |
| Private sector |  |  |  |  |  |  | √ |  |  |

† Civil society organizations and unions include: unions (Trade, Women, Farmers, and Youth), societies (Veterans, Teachers, Elderly), and religious, village and tribe leaders.

‡ International Organizations, donors include: UNICEF, WHO, FAO, World Bank, other development bank (e.g., ADB), governments of other countries (e.g., USAID, Australian Aid, UK Aid), Foundations (e.g., Bill & Melinda Gates Foundation), research foundations, international non-Governmental Organizations (NGOs), and in-country donors.
